# Neuroprotective Effect of Carnosine Is Mediated by Insulin-Degrading Enzyme

**DOI:** 10.1021/acschemneuro.2c00201

**Published:** 2022-04-26

**Authors:** Alessia Distefano, Giuseppe Caruso, Valentina Oliveri, Francesco Bellia, Diego Sbardella, Gabriele Antonio Zingale, Filippo Caraci, Giuseppe Grasso

**Affiliations:** † Department of Chemical Sciences, University of Catania, Viale Andrea Doria 6, Catania 95125, Italy; ‡ Department of Drug and Health Sciences, University of Catania, Viale Andrea Doria 6, Catania 95125, Italy; § Oasi Research Institute-IRCCS, Via Conte Ruggero 73, Troina 94018, Italy; ∥ Institute of Crystallography, CNR, Via Paolo Gaifami 18, Catania 95126, Italy; ⊥ IRCSS-Fondazione Bietti, Rome 00198, Italy

**Keywords:** diabetes, Alzheimer’s disease, insulin, IDE, carnosine, neuropeptides

## Abstract

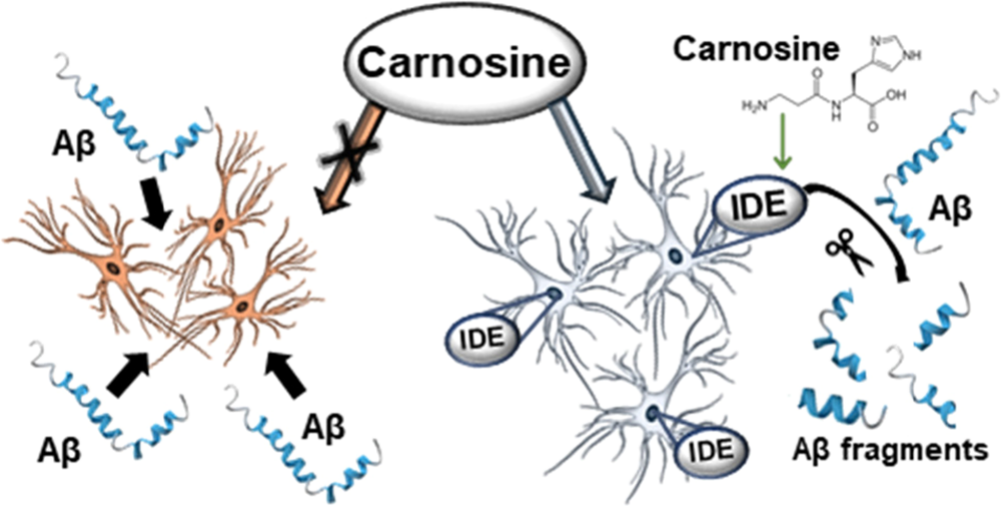

l-Carnosine is an endogenous dipeptide that has high potential for therapeutic purposes, being an antioxidant with metal chelating, anti-aggregating, anti-inflammatory, and neuroprotective properties. Despite its potential therapeutic values, the biomolecular mechanisms involved in neuroprotection are not fully understood. Here, we demonstrate, at chemical and biochemical levels, that insulin-degrading enzyme plays a pivotal role in carnosine neuroprotection.

## 1 Introduction

Amyloid ß-protein (Aβ) is a complex mixture of peptides of 37–43 amino acids in length that is present in the brain and the cerebrospinal fluid of human beings.^1^ Aβ represents the key peptide in the pathogenesis of Alzheimer’s disease (AD), a neurodegenerative disorder with a growing prevalence on a global scale. Although AD pathogenesis is not fully characterized yet, systemic accumulation of biomarkers of redox unbalance^2^ and inflammation,^3^ along with the deposition of insoluble proteinaceous aggregates in the brain, are hallmarks of disease progression.^4−6^

In the AD brain, Aβ, which is released upon enzymatic digestion of APP precursor protein in the monomeric state, typically enters a neurodegenerative pathway, which causes it to undergo aggregation characterized by the formation of larger and heavier species,^7^ among which the soluble oligomers, which anticipate the formation of insoluble aggregates, are supposed to represent the most toxic ones.^8^

Both peripheral macrophages and microglia, the brain-resident immune cells, represent two different specialized cell types activated during the immune response.^9,10^ There is a bidirectional cross-talk between microglia and neurons; in fact, neurons inform microglia regarding their status and control activation and the motility of microglia, while microglial cells are able to modulate neuronal homeostasis.^11^ Reactive microglia co-localize with Aβ within the neuritic plaques observed in the brain of AD subjects and could be implicated either in the removal or, paradoxically, in the formation of amyloid plaques.^12−15^ Microglia can promote Aβ clearance through different mechanisms including the internalization and degradation of the peptide through the endosome/lysosome pathway^16^ and the secretion of enzymes able to degrade Aβ such as insulin-degrading enzyme (IDE),^17,18^ a major enzyme responsible for the degradation of insulin (Ins) and Aβ in vitro and in vivo.^19−22^ In fact, despite Ins being the preferred substrate for IDE, the enzyme also cleaves different amyloidogenic peptides such as amylin^23^ and Aβ.^24,25^ The latter,^26,27^ as well as IDE itself,^28^ represents a well-recognized neurobiological link and a common pharmacological target between AD and type 2 diabetes (T2DM). Mice with the homozygous deletion of the IDE gene (IDE^–/−^) and an IDE deficiency show increased cerebral accumulation of endogenous Aβ, as well as hyperinsulinemia and glucose intolerance, hallmarks of T2DM.^29^ Positive allosteric modulators of the activity of IDE are currently studied as potential drugs for both pathologies, as shown by the use of a novel IDE inhibitor in a mouse model of T2DM, while activators of IDE have been considered for AD treatment.^29^ In addition, IDE has been recognized to be involved in many other biochemical pathways wherein it does not play a proteolytic action but rather a regulatory one,^19,30−33^ envisaging a multifaceted role of this enzyme within the dynamics of metabolism of living organisms.

l-Carnosine (Car) is a naturally occurring dipeptide synthesized by Car synthase composed of β-alanine and l-histidine,^34,35^ and it is highly concentrated in muscle and brain tissues. The concentration of this dipeptide is very high in cardiac and skeletal muscles, up to 20 mM,^36,37^ and remains in the millimolar range in the brain.^38^ Different studies have shown the therapeutic potential of Car in diseases characterized by abnormal oxidative stress,^39^ inflammation,^40^ and abnormal protein aggregation, such as diabetes,^41,42^ AD, but also retinal diseases.^43^ Nevertheless, the well-documented antioxidant, anti-inflammatory, and anti-aggregative activities of Car make this molecule very attractive for drug discovery approaches in neurodegenerative diseases.^44^ In addition, Car has shown the ability to interact with macrophage receptors,^45^ stimulating the phagocytic activities of these cells.^46,47^ These modulatory activities along with its ability to decrease oxidative stress and inflammation in two in vitro models (macrophages and microglia) of Aβ-induced stress^47,48^ make Car a very attractive pharmacological tool in the context of AD pathology. Indeed, in the context of AD, Car has been able to revert oxidative stress and microglial activation in a transgenic mouse model of AD,^43^ while its supplementation was shown to counteract cognitive decline in AD subjects.^49^ It is worth mentioning that the plasma concentration of Car in subjects with a presumptive diagnosis of AD is significantly lower than that detectable in age- and sex-matched healthy subjects.^50^

In the present study, we wondered whether Car can exert neuroprotective effects against Aβ oligomers through the modulation of IDE activity. We first investigated the toxic potential of Aβ1–42 oligomers in the absence or presence of Car and/or a highly selective IDE inhibitor (6bK). We conducted these studies in primary mixed neuronal cultures as a well-known and established in vitro model to study Aβ toxicity as well as the therapeutic potential of molecules of interest.^48,51^ Once the neuroprotective activity of Car was established to be IDE-mediated, we then investigated Car/IDE interaction and the molecular mechanisms underlying the protective effects of Car. For this purpose, we have applied high-performance liquid chromatography–mass spectrometry (HPLC-MS), surface plasmon resonance (SPR), dynamic light scattering (DLS), and fluorescent methods to determine the effect of Car on IDE activity, oligomerization, and cooperativity. Results indicate that the neuroprotective effect of Car is due to a modulation of IDE activity and oligomerization.

## 2 Results

### 2.1 Car Prevents the Toxicity of Aβ1–42 Induced in Mixed Neuronal Cultures via IDE

**Figure 1:**
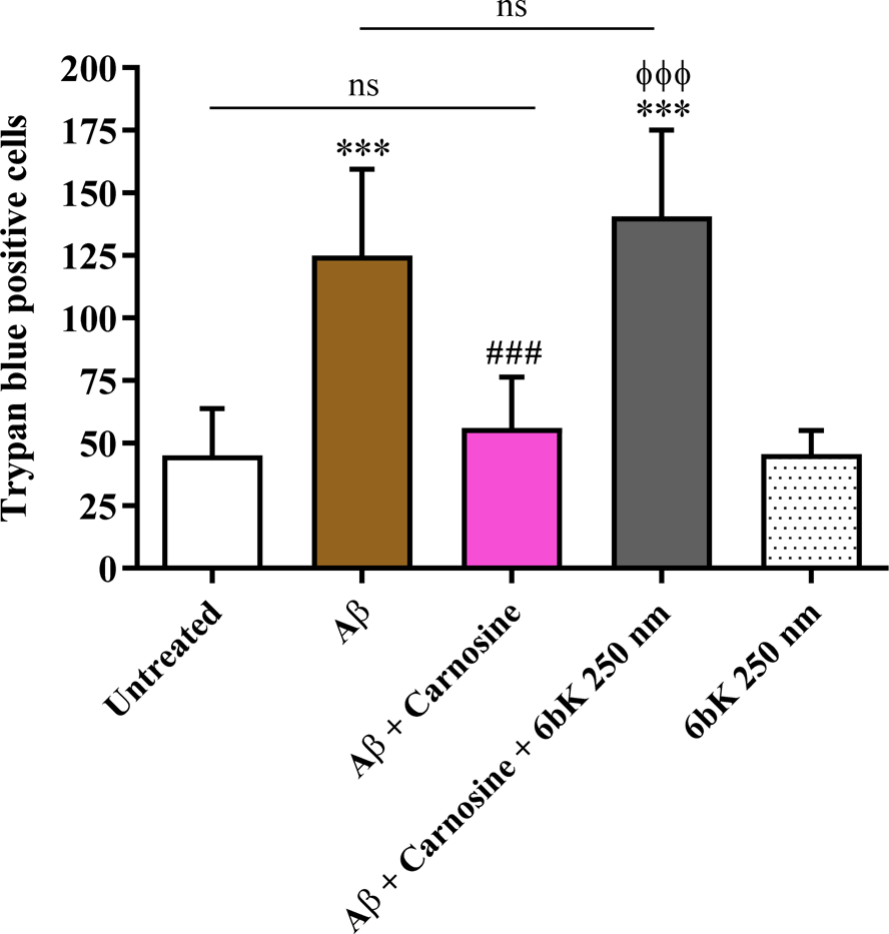
Neuroprotective effects of Car against the toxicity induced by Aβ1–42 oligomers are mediated by IDE. Primary mixed neuronal cultures were treated with Aβ1–42 oligomers (1 μM) for 48 h in the absence or presence of Car (10 mM). The effect of 6bK (highly selective IDE inhibitor) pretreatment (1 h; 250 nM) on the neuroprotective activity of Car against Aβ1–42 oligomer-induced toxicity is also shown. The toxicity of Aβ1–42 oligomers in mixed neuronal cultures was assessed by cell counting after trypan blue staining. Cell counts were performed in three to four random microscopic fields/well. Data are the mean of 7 to 8 determinations. Standard deviations are represented by vertical bars. ***Significantly different from untreated cells, *p* < 0.001, ^###^significantly different from Aβ1–42 oligomers, *p* < 0.001, ^ϕϕϕ^significantly different from Aβ1–42 oligomers + Car, *p* < 0.001; ns = not significant.

We investigated the neuroprotective activity of Car in mixed cultures of cortical cells consisting of neurons (35–40%) and glial (astrocytes and microglia; 60–65%) cells treated with Aβ1–42 oligomers (1 μM) for 48 h. Because Aβ1–42 is able to potentiate glutamate toxicity,^52^ the experiments were carried out in the presence of a cocktail of ionotropic glutamate receptor antagonists [MK-801 (10 μM) and DNQX (30 μM)] to exclude the contribution of endogenous excitotoxicity to the overall process of neuronal death. Using this model, neurotoxicity of Aβ oligomers showed faster kinetics, with a substantial increase (about 250%) in the number of trypan blue positive cells (dead neurons) being detected after 48 h of exposure to Aβ1–42 oligomers compared to untreated cells (*p* < 0.001) (Figure 1). Car significantly decreased the toxicity due to Aβ1–42 oligomers treatment in mixed neuronal cultures, giving a number of dead cells comparable to that observed for untreated cells. The highly selective IDE inhibitor, 6bK, prevented the neuroprotective activity of Car directly applied to mixed neuronal cultures treated with Aβ1–42 oligomers (*p* < 0.001 compared to Aβ1–42 oligomers + Car). Thus, these experiments show for the first time that IDE is implicated, at least in part, in mediating the neuroprotective effects of Car. The treatment with 6bK had no effect *per se* on mixed neuronal culture viability in the absence of Aβ1–42 oligomers.

An additional set of experiments in which mixed neuronal cultures were treated with Aβ1–42 oligomers for 48 h in the absence or presence of increasing concentrations of 6bK (100 and 250 nM) was carried out. The results reported in Figure 1S show how the selective IDE inhibitor did not significantly modify the Aβ-induced cell death.

In order to understand the role played by glial cells in the neuroprotective effects of Car, we treated primary pure neuronal cultures with Aβ1–42 oligomers for 48 h in the absence or presence of Car. As shown in Figure 1S, the treatment of pure neurons with Aβ1–42 oligomers significantly decreased cell viability compared to untreated cells (*p* < 0.001). Differently from mixed neuronal cultures, Car was not able to revert the toxic effects mediated by Aβ1–42 oligomers, underlining the key role played by glial cells in the neuroprotection elicited by Car.

### 2.2 Car Induces IDE Oligomerization

**Figure 2:**
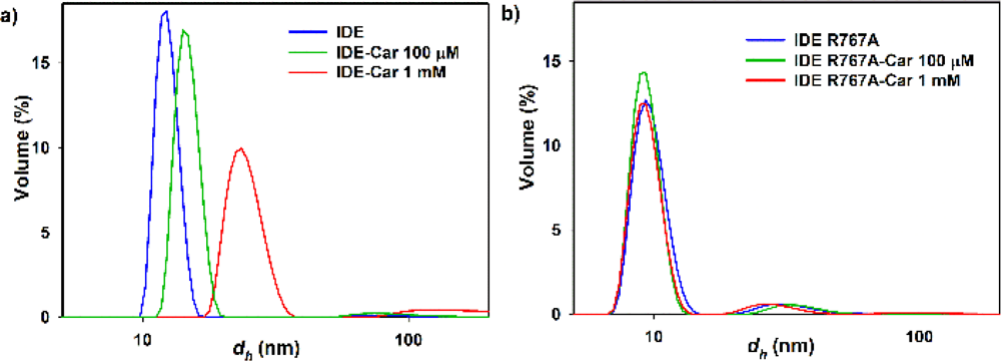
DLS measurements of (a) IDE wild type and (b) IDE R767A in the presence of increasing concentrations of Car.

DLS was performed to determine the average hydrodynamic diameter (*d*_h_) of IDE in the presence of Car. A dose-dependent effect of Car on the stability, conformation, and aggregation of IDE was revealed through thermal denaturation experiments (see Supporting Information). IDE alone showed a hydrodynamic diameter of 12.7 ± 0.9 nm, a finding consistent with previous studies.^53^ At a concentration of 1 mM, Car caused an increase of IDE diameter (22 ± 2 nm), suggesting that an oligomerization process of IDE actually occurred (Figure 2). DLS measurements were also performed using IDE R767A. This mutation hinders the oligomerization properties of IDE and, therefore, IDE R767A is mainly monomeric. The hydrodynamic diameter of the IDE mutant was found to be 10 ± 1 nm, supporting a minor presence of oligomeric species in solution.^32,54^ DLS data showed that Car did not significantly affect the hydrodynamic diameter of the IDE R767A. This result demonstrates that Car could induce the oligomerization of IDE.

**Figure 3:**
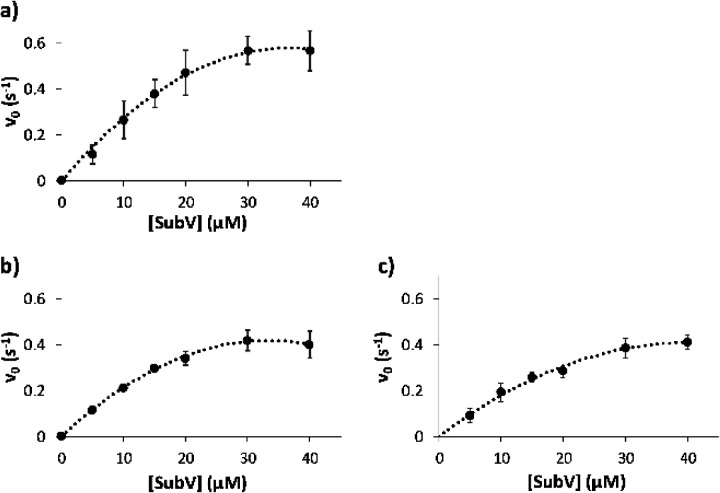
Kinetic graphs related to the IDE-mediated hydrolysis of substrate V (a) also in the presence of Car 0.1 mM (b) or 1 mM (c).

In addition, the thermal denaturation of IDE was also followed to study the effects of Car on the stability, conformation, and aggregation of IDE at pH 7.4. Figure 3S shows the derived count rate (DCR) variation when the temperature of an IDE solution was increased in the presence/absence of Car. The DCR represents the scattering intensity measured at the detector in the absence of the laser light attenuation filter and therefore it is related to both size and concentrations of the protein.^55^ As for IDE alone, DCR increased over 50 °C in DLS experiments upon the protein denaturation. Car at 0.1 mM appeared to slightly anticipate the denaturation process, whereas at 1 mM, it promoted a change of IDE size at low temperature. These data further support a dose-dependent effect of Car on IDE stability and oligomerization.

### 2.3 Car Differently Modulates IDE Activity In Vitro toward Long and Short Substrates

The activity of IDE has been tested for different substrates and by different methods. Indeed, it is well known that IDE activity and allosteric mechanisms are different depending on the length of the substrates, as only long substrates are able to bind the catalytic site as well as the exosite.^56^ For this reason, Car has been tested as a possible IDE activator toward the degradation of Ins, Aβ1–40, and the short fluorogenic peptide, substrate V.^53^ In Figure 4S, the cleavage sites of IDE on Ins and Aβ1–40 as obtained by HPLC-MS detection of the peptide fragments generated by the incubation with IDE are reported, whereas in Figure 5S, the normalized areas of the peaks assigned to intact Ins and Aβ1–40 in absence and presence of Car 100 μM and 1 mM as a function of incubation time are plotted.^23^ In both cases, Car enhances peptide degradation by IDE as the decrease of the Ins and Aβ1–40 molecular peaks is more rapid in the presence of the dipeptide. For this reason, MS experiments confirmed that Car is an activator of IDE activity toward both long substrates tested, Ins and Aβ1–40.

As for the IDE activity toward a short substrate, Figure 3 shows the IDE kinetic graphs, obtained by plotting the initial velocity as a function of the substrate V concentration (a), also in the presence of different amounts of Car (b, c). Based on these results, Car seems to slightly lower the maximum reaction rate, suggesting that Car affects the IDE-dependent degradation of substrate V in a noncompetitive manner. This effect of Car on the IDE activity toward substrate V, different from that reported for that on the IDE-mediated hydrolysis of both Ins and Aβ1–40, could reasonably be ascribed to a different substrate dimension. Bearing that in mind, the fluorimetric assay results showed that Car did not act as an IDE activator toward short peptides like it does for longer ones.

Finally, it is important to highlight that, besides Car addition, the pH and all the other experimental values have been kept constant during all activity measurements. In order to check if the Car effect was molecule-specific, we have performed IDE activity modulation experiments in the presence of d-carnosine and carnitine, using Ins as a substrate. In neither cases, IDE activity modulation was observed (data not shown), demonstrating a specific modulatory activity for Car and the selective role of stereochemistry.^57^

### 2.4 Car Increases IDE Cooperativity

It has been reported that IDE has a Hill coefficient for the degradation of small fluorogenic substrates equal to about 2.0.^58^ In this work, we have measured IDE-Ins interaction kinetic parameters in the presence and in the absence of Car at different concentrations, by applying a novel SPR approach described in the Supporting Information. In Table 1, the obtained results are reported for both wild-type and IDE R767A. It is possible to see that the effect of Car on the Hill coefficient value *n* (second column of Table 1) is remarkable. Interestingly, such effect is dependent on the concentration of Car and is not detected in the case of IDE R767A, confirming the DLS results. The variation of the *K*_D_ value observed in the presence of Car in the case of IDE R767A indicates that a contribution to the activation of the enzyme is possibly given also to a Car induced higher affinity of IDE toward Ins.

**Table 1:** Hill Coefficient (*n*) and the Dissociation Constant (*K*_D_) Extrapolated from the SPR Analysis of IDE-Ins and IDE R767A-Ins in the Absence and Presence of Car^a^

solution	*n*	*K*_D_	*R*^2^
IDE-Ins	1.89 ± 0.10	1.52 × 10^–5^ ± 1.19 × 10^–6^	0.9999
IDE-Ins + Car 100 μM	2.26 ± 0.21	1.70 × 10^–5^ ± 2.16 × 10^–6^	0.9998
IDE-Ins + Car 1 mM	3.36 ± 0.65	8.09 × 10^–6^ ± 5.79 × 10^–7^	0.9977
IDE R767A-Ins	1.24 ± 0.03	2.66 × 10^–1^ ± 7.91 × 10^–2^	0.9987
IDE R767A-Ins + Car 100 μM	1.25 ± 0.02	1.25 × 10^–4^ ± 3.07 × 10^–5^	0.9993
IDE R767A-Ins + Car 1 mM	1.31 ± 0.53	3.50 × 10^–5^ ± 6.07 × 10^–5^	0.9980

a The *R*-square is also reported in the last column.

## 3 Conclusions

As experts in the field continue to advertise, “many of the most exciting new possibilities hinge on the development of powerful pharmacological modulators of IDE.”^59^ Car is an endogenous peptide that can be also given orally as a beta-alanine supplement, widely used by many people, especially athletes to improve their performances.^60^ Although the presence of Car in the serum is not detectable because of its rapid degradation by serum carnosinase,^61^ intact Car is excreted in urine up to 5 h after intake, indicating that the dipeptide resists somehow to degradation.^62^ As it is widely reported that Car is neuroprotective,^63^ here we have explored the possibility that Car exerts its beneficial effect through the modulation of IDE. Our results obtained in rat mixed neuronal cultures clearly show that Car is protective against Aβ1–42-induced toxicity and also that the neuroprotective activity of Car is lost in the presence of 6bK, a highly selective IDE inhibitor, supporting the recent findings described by Fu et al.^18^ demonstrating that microglia partially degrade Aβ via the secretion of IDE. In order to understand the molecular basis of such an intriguing result, we have applied various experimental approaches to assess the Car mechanism of action on IDE. Indeed, DLS measurements show that Car alters the average hydrodynamic radius of the enzyme, hinting to higher oligomeric forms induced by the presence of Car in a concentration-dependent manner. We exclude that the change in the hydrodynamic radius could be due to a change in enzyme conformation, as IDE R767A used to test such hypothesis did not show the same trend. In accordance to this result, SPR measurements applied to calculate the Hill coefficient gave a clear indication that Car directly affects the enzyme cooperativity, increasing the value of the Hill coefficient in a concentration-dependent manner. Last but not least, HPLC-MS experiments clearly show an increase in IDE activity toward both Ins and Aβ peptides in the presence of Car. On the contrary, the IDE degradation of a smaller fluorogenic substrate does not seem to be affected by the presence of Car. The latter findings give a clear indication on the possible mechanism involved in Car neuroprotection. Indeed, all results point at an IDE activating role of Car due to an increase in the oligomerization and in the cooperativity of the enzyme, which increase the enzyme capability to degrade long substrates such as Ins and Aβ peptides, but not shorter one such as substrate V. This specific regulatory mechanism indicates that Car does not bind to the IDE catalytic site, being a heterotropic modulator, as it is able to regulate the enzyme activity by binding to the exosite or to other not identified sites, causing a different interaction between the enzyme and long substrates, changing their reciprocal affinity and, in turn, IDE catalytic activity. Such a result is in accordance with previous findings already reported for IDE activity^53,56^ and opens a new path to explore the therapeutic potential of Car in AD.

## Supporting Information Available

Materials and reagents; preparation of Ab1–42 oligomers; primary pure and mixed neuronal cultures; measurement of cell viability and cell death by the MTT and trypan blue exclusion assays; statistical analysis; study approval; dynamic light scattering (DLS) measurements; HPLC-MS; enzymatic assays; and surface plasmon resonance (PDF).

## Abbreviations

IDE: insulin-degrading enzyme
Ins: insulin
Car: l-carnosine
AD: Alzheimer’s disease
PD: Parkinson disease
HPLC-MS: high performance liquid chromatography-mass spectrometry
SPR: surface plasmon resonance
DLS: dynamic light scattering
T2DM: type 2 diabetes.
