# Supplementary material for: Neuroprotective Effect of Carnosine Is Mediated by Insulin-Degrading Enzyme
Source: ACS Chem Neurosci. 2022 Apr 26;13(10):1588–93. doi: 10.1021/acschemneuro.2c00201 (PMC9121383; doi:10.1021/acschemneuro.2c00201)
Supplement: Supplementary file 1 — cn2c00201_si_001.pdf [file cn2c00201_si_001.pdf]

Supplementary Information

The neuroprotective effect of carnosine is mediated by insulin-degrading enzyme

Alessia Distefano,[1‡] Giuseppe Caruso,[2,3‡] Valentina Oliveri,[1] Francesco Bellia,[4] Diego Sbardella,[5] Gabriele Antonio Zingale,[1] Filippo Caraci[2,3*] and Giuseppe Grasso[1*]

1 Department of Chemical Sciences, University of Catania, Viale Andrea Doria 6, 95125, Italy

2 Department of Drug and Health Sciences, University of Catania, Viale Andrea Doria 6, 95125, Catania, Italy

3 Oasi Research Institute - IRCCS, Via Conte Ruggero 73, 94018, Troina, Italy.

4 Institute of Crystallography, CNR, Via Paolo Gaifami 18, 95126, Catania, Italy

5 IRCSS-Fondazione Bietti, Rome, Italy.

[*] fcaraci@unict.it

[*] grassog@unict.it

Materials and reagents

Cultures of pure and mixed cortical neurons were obtained from rats at embryonic day 15 (Harlan Laboratories, Italy). Neurobasal medium, B27 supplement, fetal calf serum (FCS), and horse serum (HS) were obtained Thermo Fisher Scientific (Waltham, MA, USA). Dulbecco's Modified Eagle Medium (DMEM), DMEM/Ham's F12, penicillin, and streptomycin were supplied by American Type Culture Collection (ATCC, Manassas, VA, USA). HFIP-treated amyloid-peptide (1-42) was obtained from Bachem Distribution Services GmbH (Weil am Rhein, Germany). The IDE inhibitor 6bK was supplied by Tocris Bioscience (Bristol, UK).

The human recombinant Insulin Degrading Enzyme (IDE) solution, 0.15 mg/mL in 20mM Tris, 200mM NaCl, 1mM DTT at pH = 8, was purchased from Giotto Biotech (Italy).

The phosphate buffered saline (PBS) stock solution was prepared dissolving one tablet (from Sigma-Aldrich) in 100 mL of ultrapure water (18.2 MΩ cm, TOC 1 pbm) obtained from PURELAB Flex 3 system. The resulting solution contains 137 mM Sodium Chloride, 2.7mM Potassium Chloride and 10mM Phosphate buffer. Prior to experiments, the PBS buffer was filtered with a non-pyrogenic syringe filter (Minisart purchased from "sartorius stedim biotech" cut-off = 0.2µM)

The human recombinant Insulin (dry powder, MW = 5807,65 g/mol CAS number 11061-68-0) was acquired from Sigma-Aldrich, and the stock solution was freshly prepared by dissolving 1.3 mg of Insulin in HCl 5mM to attain a final concentration of 0.2mM.

The 3.3'-Dithiodipropionic acid (N-hydroxysuccinimide ester) (Lomant's reagent, $C_{14}H_{16}N_2O_8S_2$, MW = 404.42 mg/mL, CAS number 57757-57-0) and the Bovine serum albumin (BSA, lyophilized powder,

crystallized, MW: 66430,3 g/mol, purity ≥ 98%, CAS number 9048-46-8) were purchased from Sigma-Aldrich.

The BSA stock solution was freshly prepared by dissolving 40.2 mg of the protein in HCl 0.5 mM to reach a final concentration of 500 µM.

The Ethanolamine-HCl (MW: 97.54 g/mol, purity ≥ 98%, CAS number 2002-24-6) and the Ethanol solution (MW: 46.07 g/mol; CAS number 64-17-5), used for the final wash step of gold sensor, were purchased from Sigma-Aldrich.

Dimethyl Sulphoxide (DMSO; MW: 78.13; purity > 99.5%; CAS number 67-68-5) was acquired from ROMIL Pure Chemistry Ltd.

Trifluoroacetic acid (TFA; MW= 114.02 g/mol; CAS number 76-05-1), Acetonitrile (CH_3CN; MW= 41.05 g/mol; CAS number 34851), Carnosine used for all experiments (β-Alanyl-L-histidine; purity ≈99%; MW: 226.23 g/mol; CAS number 305-84-0) were purchased from Sigma Aldrich.

All Surface Plasmon Resonance experiments were performed by using a multiparametric Bionavis SPR Navi 210A instrument. The latter is equipped with two separate and parallel channels (reference and sample channel) as well as two distinct lasers at two wavelengths: 670 nm and 750nm. For our experiments, the wavelength used was 670nm. Experiments were run on a SPR102- AU gold sensor chip. This latter is composed by 50nm of gold, 2nm of chromium (for adhesion) on glass substrate. This substrate is 20mm long, 12 mm wide and 0.55 mm thick, whereas the area where the enzyme is immobilized is 12 mm^2.

The chromatographic system was the Finnigan Surveyor Plus HPLC System, equipped with a Jupiter 5 µm C4 300 Å column produced by Phenomenex.

The mass analyses have been conducted with a Finnigan LCQ Deca XP Max LC / MSn (Thermo Electron Corporation, USA), with an ESI source and an ion trap analyser, capable of scanning a m/z range from 15 to 4000 with a nominal resolution of 10000 Normal Unit mass scan. Moreover, the capillary temperature was 300 °C.

The incubator (I-Cell 55 lt. standard Blue-Line; sil.mar. instruments s.r.l) in which the reaction occurs is equipped with a temperature control system.

All the data collected during the analyses were processed through TraceDrawer software® and Xcalibur™ for SPR measures and HPLC-MS respectively and Origin software for all data analysis.

Preparation of Aβ1-42 Oligomers

The oligomers of Aβ1-42 were prepared starting from the monomeric form of the peptide as previously described.[1,2] Briefly, the monomeric form of lyophilized HFIP-treated Aβ1-42 was first

suspended in DMSO at the final concentration of 5 mM and diluted in ice-cold cell culture medium DMEM/F12 (1:1) at the final concentration of 100 μM. Aβ1-42 samples were then incubated at 4 °C for 72 hours and immediately used to treat primary pure or mixed neuronal cultures or aliquoted and stored at -20 °C until their use.

Primary pure and mixed neuronal cultures

Cultures of pure cortical neurons were obtained from rats at embryonic day 15 as previously described.[3] Briefly, cortical cells obtained from cortices dissection were plated on 48-well plates pre-coated with 0.1 mg/ml Poly-D-lysine at a density of 2 × 10^5/well. Neurons were grown in Neurobasal medium supplemented with B27, glutamine (2 mM), and the antibiotics penicillin (100 U/ml) and streptomycin (100 μg/ml). In order to avoid any proliferation of non-neuronal cells, Cytosine-D-arabinofuranoside (10 μM) was added to each dish 18 hours after plating and was kept for 3 days before the next change of medium. As confirmed by using immunocytochemistry for glial fibrillary acidic protein and flow cytometry for neuron-specific microtubule-associated protein 2, this method allows to obtained neuronal cultures with a very high purity (>99%).[1,4]

Primary mixed neuronal cultures (35–40% of neurons; 60–65% of astrocytes and microglia) were obtained from rats at embryonic day 15 as previously described.[1,3] Cells were plated at a density of 2 × 10^5/well on 48-well plates and grown into MEM supplemented with HS (10%), FCS (10%), glutamine (2 mM), and glucose (6 mg/ml). In order to avoid any proliferation of non-neuronal cells, Cytosine-D-arabinofuranoside (10 μM) was added to each dish 5 days after plating and was kept for 3 days before the next change of medium.

Measurement of cell viability and cell death by the MTT and trypan blue exclusion assays

Pure neuronal cultures were treated at 7 days *in vitro* with Aβ1-42 oligomers (1 μM) for 48 hours both in the presence and in the absence of carnosine (Car) (10 mM). Neuronal cell viability was measured by the MTT ([3-(4,5-dimethylthiazol-2-yl)-2,5-diphenyltetrazolium bromide]) assay. At the end of the treatment cells were incubated with MTT (0.9 mg/ml final concentration) for 2 hours at 37 °C. Following this incubation step, the MTT solution was removed and the formazan crystals were melted by using DMSO. As a last step, 200 µL coming from each type of sample were transferred to a 96-well plate and the absorbance at 569 nm was read using a plate reader (Spectra Max M5, Molecular Devices, Sunnyvale, CA, USA). Untreated neuronal cells were used as controls.

In the case of primary mixed neuronal cultures, cells were treated with Aβ1-42 oligomers (1 μM) for 48 hours both in the presence or in the absence of Car (10 mM). The possible neuroprotective activity against Aβ1-42-induced toxicity played by IDE was indirectly investigated by using the specific inhibitor of this enzyme, 6bK, at the final concentration of 250 nM. At the end of the treatment, the toxicity induced

by Aβ1-42 oligomers and the neuroprotection exerted by Car were quantitatively assessed by trypan blue exclusion assay.[1] Cell counts was performed in three to four random microscopic fields/well.

2.5. Statistical analysis

Graphpad Prism (Ver. 8, San Diego, CA, USA) software was used for statistical analysis. For multiple comparisons, one-way ANOVA followed by Bonferroni's *post hoc* test was employed. For all the experiments, the statistical significance was set up at p values lower than 0.05.

2.6. Study Approval

The study in neuronal cultures was authorized by the Institutional Animal Care and Use Committee (IACUC) of the University of Catania (OPBA Project #169). Animal care followed Italian (D.M.116192) and EEC (O.J. of E.C. L 358/1 12/18/1986) regulations on protection of animals used for experimental and scientific purposes.

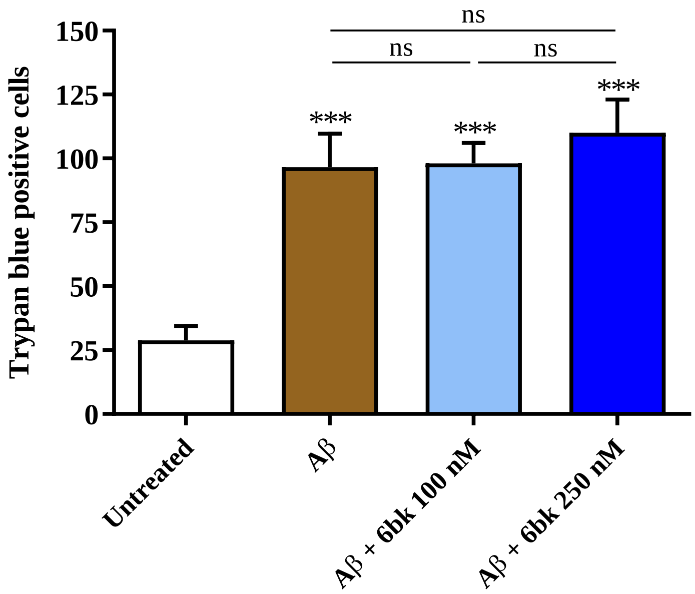

Figure 1S. Change in the cell death caused by treating primary mixed neuronal cultures with Aβ1-42 oligomers (1 µM) for 48 hours, in the presence and in the absence of 6bK (100 or 250 mM). The cell death under the different experimental conditions was assessed by cell counting after trypan blue staining. Cell counts was performed in three to four random microscopic fields/well. Data are the mean of four determinations. Standard deviations are represented by vertical bars. ***Significantly different from untreated cells, $p < 0.001$; ns = not significant.

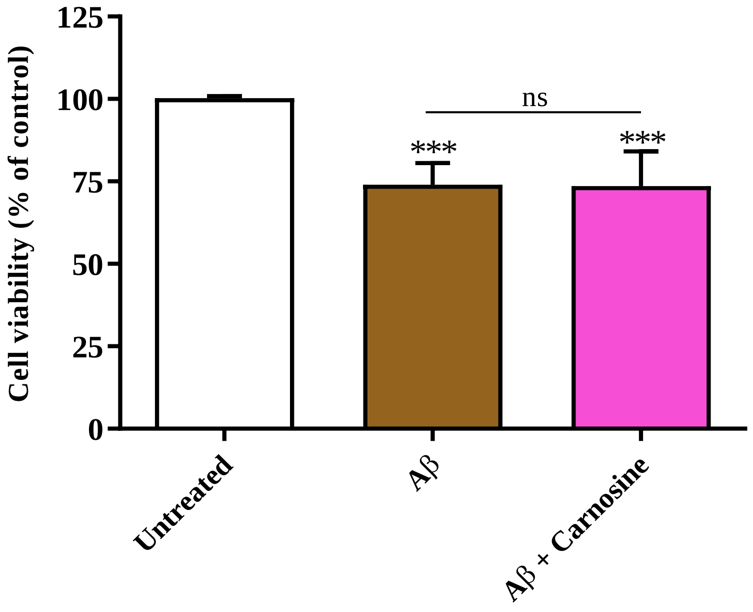

Figure 2S. Change in the cell viability (measured by the MTT assay) caused by treating primary pure neuronal cultures with Aβ1-42 oligomers. Pure neuronal cultures cells were treated for 48 hours with Aβ1-42 oligomers (1 µM), in the presence and in the absence of Car (10 mM). Data are the mean of 9 determinations and are expressed as the percent variation with respect to the viability recorded in untreated (control) cells. Standard deviations are represented by vertical bars. ***Significantly different from untreated cells, $p < 0.001$; ns = not significant.

Dynamic light scattering measurements

The measurements were performed with a Zetasizer Nano ZS (Malvern Instruments Ltd., UK) instrument equipped with a He–Ne laser. DLS measurements were run on disposable, temperature-resistant microcuvettes by using optimal measurement times and laser attenuation settings. All samples were measured 4-5 times with at least 12 accumulated scans. The sample solution was filtered through a 0.22 μm filter before the beginning of each experiment. The thermal denaturation of IDE was followed by DLS in the presence/absence of Car. IDE or IDE R767A (1.3 μM) was incubated alone or in the presence of Car (0.1 and 1 mM) in Tris buffer (pH 7.4). DLS data were acquired from 25 to 65 °C at 2 °C intervals with an equilibration time of 180 s.

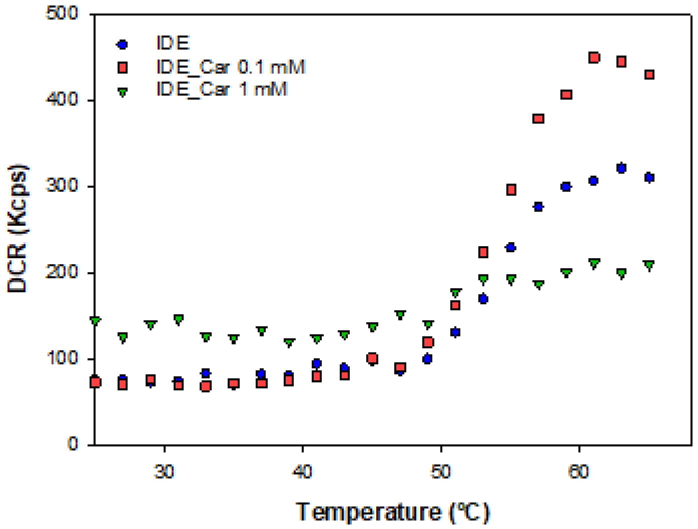

Figure 3S. Representative DCR trends as a function of the temperature of IDE and mixtures of IDE/Car (pH 7.4)

HPLC – Mass Spectroscopy

The enzymatic digestions of Human Ins and Aβ_{1-40} were performed according to the following methods: A solution of Ins (20μM) or Aβ_{1-40} was incubated with IDE (180nM) in PBS buffer solution (pH 7.3) at 37°C inside a controlled temperature incubator. The experiment was monitored over time, in the absence or in the presence of Car 100 μM and 1mM. Lastly, 10% TFA was added for each sample in order to quench the IDE activity. The TFA final concentration was the aliquots is 0.2%.

The kinetic degradation of Ins and Aβ_{1-40} and the analysis of their fragments have been obtained through the use of HPLC-Mass Spectrometry (See Figure 4S and Table 1S). The mobile phase consists in a mixture of high purity water (Milli-Q Element Ultrapure Water) and high purity acetonitrile (CH_3CN). Moreover, the mobile phase was degassed for 30 minutes. To this solution, TFA was added to both solvents until it reached a concentration of 0.05%. The use of TFA is crucial in chromatography because it is used as an ion-pairing agent allowing the suppression of the rejection of residual silanols of the stationary phase, thus hindering nonspecific interactions between the analytes and the stationary phase.[5] In Figure 5S the intensities of Ins and Aβ_{1-40} peaks expressed as normalized area are reported respectively, calculated as follows:

(Area peptide t/ Area tot t) *100

where *Area peptide t* is the area of the Ins or Aβ_{1-40} peak per incubation time *t* and *Area tot t* is the sum of all peaks for each incubation time (0 min; 10min; 45min; 60min; 180min for Ins digestion and 0 min; 10 min; 30min; 45 min for Aβ_{1-40} digestion). During the analysis, the chromatographic column was kept at a temperature of 34°C, with a workflow of 250 μL/min and each experiment lasted 40 min with a starting waste of time of 18 minutes.

Ins Fragment	Retention time (min)	Observed Peaks (m/z)	Calculated Peaks (m/z)
[A (1-14) B (1-16)]	28.76-28.83	1117.80	1117.80
[A (14-21) B (10-30)]	28.21-28.43	1163.35	1163.40
[A (1-13) B (1-9)]	26.98-27.24	1179.08	1179.80
[A (1-13) B (1-10)]	26.58-26.78	1246.98	1248.90
[A (1-14) B (1-9)]	27.38-27.52	1259.70	1261.40

$A\beta_{1-40}$ Fragment	Retention time (min)	Observed Peaks (m/z)	Calculated Peaks (m/z)
1 - 19	24.41	1158.39	1158.24
20 – 40	27.67	943.73	944.09
21 - 40	28.04	1017.39	1017.68
29 - 40	28.55	1085.56	1085.36

Table 1S. Summary of MS analyses of Ins and $A\beta_{1-40}$ fragments obtained by IDE digestion and used for a semi-quantitative estimation of enzyme activity.

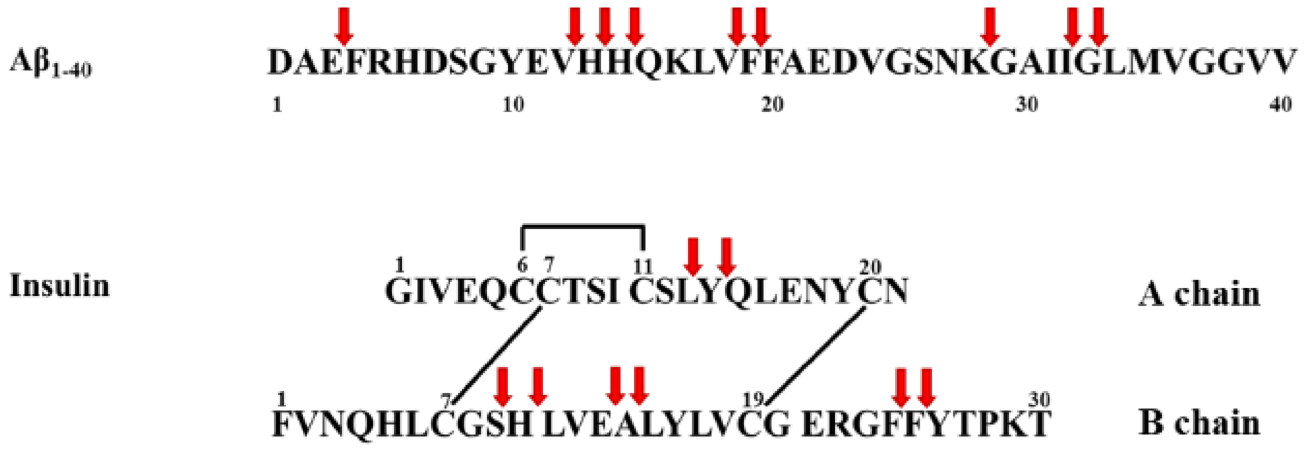

Figure 4S. cleavage sites of IDE on Aβ1-40 and Ins detected by HPLC-MS. The relative intensities of the generated peptide fragments have been used to assess IDE activity changes in the presence of Car.

a)

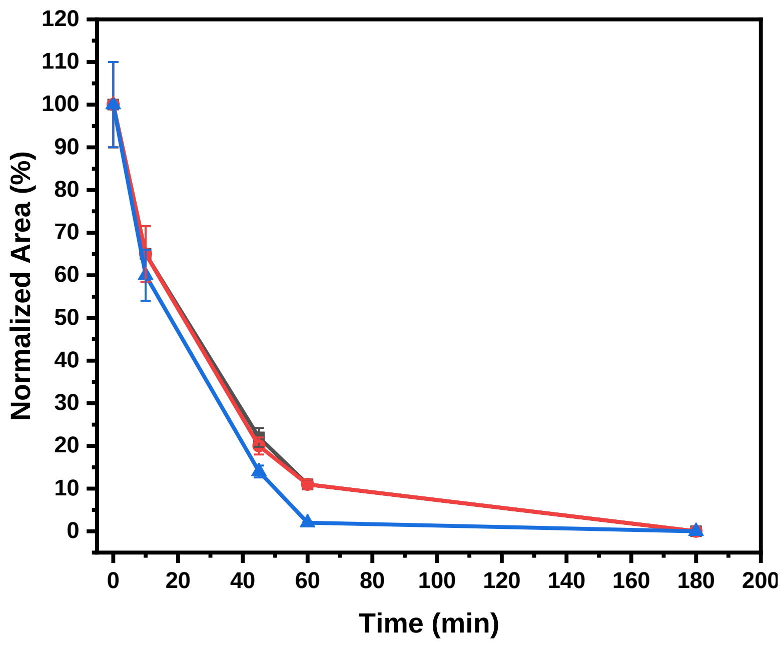

b)

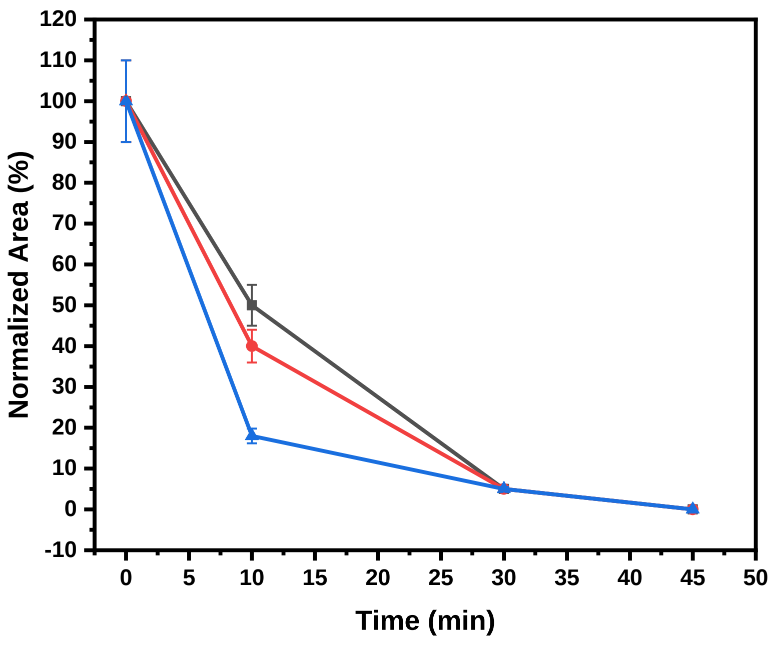

Figure 5S. Normalized areas of molecular peak of a) Ins and b) Aβ peptide in absence (dark grey) and in presence of Car 100µM (red) and 1mM (blue) at the indicated times of incubation.

Enzymatic assays

Reactions were carried out at 37 °C, using the different concentrations (0-40 µM) of substrate V (7-methoxycoumarin-4-yl-acetyl-RPPGF-SAFK-2,4-dinitrophenyl; R&D Systems), in the presence of rat IDE (Sigma-Aldrich) (50 nM) in PBS (pH 7.4). The same conditions were used to assess the activity of IDE towards substrate V in the presence of Car 0.1 mM and 1 mM. Cleavage of substrate V was assessed by monitoring the fluorescence increase for 3 min at 20-s intervals on a microplate reader (Varioskan Flash, Thermo Fisher), the excitation and emission wavelength values being 320 nm and 405 nm, respectively. It is important to highlight that Car did not affect the overall fluorescent response, as it is reported in Figure 6S.

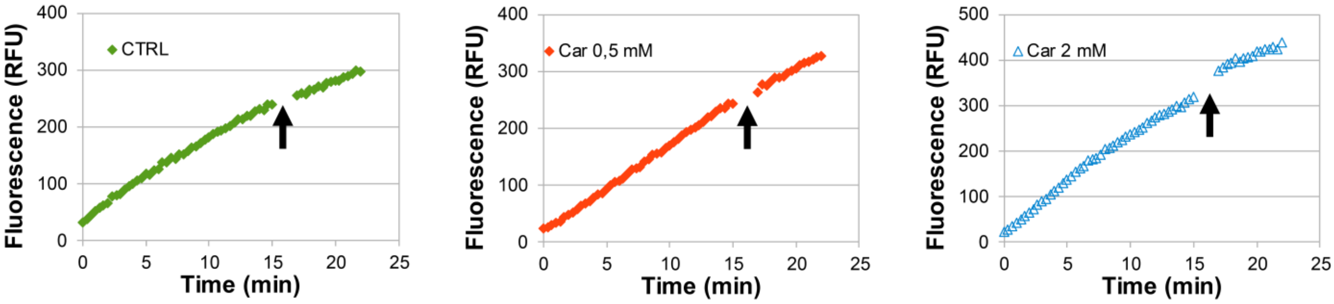

Figure 6S. Car effect on the fluorimetric assay. Car was added (↑ time of Car addition) at the concentrations indicated ([PBS buffer] = 10mM, [KCl] = 2.7 mM, [NaCl] = 137mM, pH = 7.4, [IDE] = 50 nM, [substrate V] = 20 µM) and did not affect the substrate V fluorescent response.

Surface Plasmon Resonance

Functionalization of gold sensor chip

The gold sensor chip was functionalized with the Lomant's reagent in DMSO (Lomant's reagent/DMSO stoichiometric ratio = 2) under a nitrogen atmosphere for five days.[6]
After five days, the sensor was washed with fresh DMSO, ultrapure water, ethanol and lastly it was dried out with a N_2 flow.

Immobilization of Insulin Degrading Enzyme (IDE)

The immobilization of IDE was carried out by fluxing a solution of 100nM of IDE in PBS (pH = 7.4) on a functionalized gold sensor chip in parallel configuration (only on the sample channel) at 15µl/min for 15 minutes.

The immobilization occurs via peptide bonds between the ammino groups of IDE and the carboxylic groups of Lomant's reagent with the release of N-hydroxysuccinimide group.
Once the immobilization of IDE was reached, a solution of Ethanolamine-HCl (1M, pH = 8) was flowed on the sensor twice in serial configuration (both sample and reference channels) at 15µl/min for 15 minutes in order to deactivate all the residual active sites on the surface.
In order to evaluate the number of IDE molecules immobilized on the surface, the SPR angle shift due to the enzyme immobilization has been measured and, in our experimental conditions, resulted to be 0.222 deg. Noteworthy, as a rule of thumb, if a wavelength of 670nm is employed, 1 ng mm^{-2} of immobilized absorbate corresponds to a signal of about 1000 RU SPR angle shift. For our instrument (Bionavis SPR Navi 210A) 1 mdeg SPR angle shift corresponds to 8.0 resonance unit (RU), therefore 222 mdeg is equal to 1776 RU. Hence the ng deposited on mm^2 corresponding to 1.776 ng.
According to the gold sensor chip used, the real portion of surface where the immobilization occurs is 12 mm^2. As a result, the ng deposited on 12mm^2 corresponds to 21.312 ng and the number of IDE molecules immobilized onto the examined surface is 9.104 x 10^{11} molecules cm^{-2}. This number is obtained by dividing the molecular weight of IDE and multiplied by the Avogadro's number.
Moreover, the adlayer thickness (*d*) of IDE was calculated from the following equation:

$$d(cm) = \frac{\theta(\text{molecules/cm2})}{N(molecules/cm2)}$$

where θ corresponds to 9.104 x 10^{11} molecules cm^{-2} and N is the bulk number density of the adsorbate. This latter is obtained by dividing the bulk density of adsorbate ρ (ρ = 1.30 g cm^{-3})[7] and the Avogadro's number multiplied by the molecular weight.
Thus, taking in account the previous formula, the IDE adlayer thickness (d) is 1.37 nm.
In order to minimize the aspecific interactions between insulin (Ins) molecules and the reference channel, a Bovine Serum Albumin (BSA) solution (100 µM) was flowed on the sensor in parallel configuration (only on the reference channel) at 30µl/min for 7 minutes of injection. Indeed, the common blocking agent used for activated ester groups is Ethanolamine, but a further treatment of the sensor with a BSA solution allows a more thorough deactivation of the reference channel surface.[8,9]
Analogously, the same procedure was applied for the immobilization of IDE monomeric variant: a solution of IDE R767A (50nM) was fluxed onto the sample channel. The SPR angle shift reached was 0.205 therefore the value of θ corresponds to 1.01 x 1011 molecules cm^{-2}, and assuming that the bulk density of adsorbate ρ (ρ = 1.30 g cm^{-3}) is the same for the monomeric variant, the IDE R767A adlayer thickness (d) resulted to be 1.30 ± 0.18 nm.

SPR Method to calculate the Hill coefficient

Human Ins in PBS buffer at five different concentrations (500nM; 2.5 µM; 5 µM; 10 µM; 20 µM) were prepared as serial dilutions starting from the Ins stock solution at concentration of 0.2mM.

All solutions were flowed onto the surface in serial configuration at 30µl/min for 7 minutes of injection time and 15 minutes of post-wait time. During the latter, the fluxing of running buffer (PBS) allows the removal of the Ins molecules from the surface after the interaction with IDE and results are obtained by subtracting the signal from the reference channel containing BSA. The analysis of all resulting curves and the extrapolation of kinetic parameters *(n,* K_D*)* were carried out by using the Origin software. In addition, the investigation of the interaction between immobilized IDE and Ins solutions was carried out also in the presence of Car at two different concentrations (100 µM; 1mM) in PBS buffer. Both sets of measurements, composed by five samples with the different Ins concentrations, have been carried out using constant Car concentrations.

Lastly, in case of IDE R767, the interactions with Ins in the absence and in the presence of Car at two different concentrations (100µM; 1mM) were also examined and affinity analyses were performed.

The maximum SPR response extrapolated from the steady state of each curves were plotted versus the Ins concentrations and the data points were fitted with the Hill's binding model function present in the Origin software. All the curves were obtained by using the following equation:

$$Y = Y_0 + (\,Y_{MAX} - Y_0) \cdot \frac{X^n}{K_M^n + X^n}$$

Y_{MAX} and Y_0 are the maximum and the minimum values of the function, respectively, X is the insulin concentration, *n* and K_M are, the Hill coefficient and the Michaelis-Menten constant, respectively. The obtained results are reported in Table 1 of the manuscript.

References

(1) Caruso, G.; Fresta, C. G.; Musso, N.; Giambirtone, M.; Grasso, M.; Spampinato, S. F.; Merlo, S.; Drago, F.; Lazzarino, G.; Sortino, M. A.; Lunte, S. M.; Caraci, F. Carnosine Prevents Aβ-Induced Oxidative Stress and Inflammation in Microglial Cells: A Key Role of TGF-B1. *Cells* **2019**, *8* (1), E64. https://doi.org/10.3390/cells8010064.

(2) Torrisi, S. A.; Geraci, F.; Tropea, M. R.; Grasso, M.; Caruso, G.; Fidilio, A.; Musso, N.; Sanfilippo, G.; Tascedda, F.; Palmeri, A.; Salomone, S.; Drago, F.; Puzzo, D.; Leggio, G. M.; Caraci, F. Fluoxetine and Vortioxetine Reverse Depressive-Like Phenotype and Memory Deficits Induced by Aβ1-42 Oligomers in Mice: A Key Role of Transforming Growth Factor-B1. *Front Pharmacol* **2019**, *10*, 693. https://doi.org/10.3389/fphar.2019.00693.

(3) Caraci, F.; Tascedda, F.; Merlo, S.; Benatti, C.; Spampinato, S. F.; Munafò, A.; Leggio, G. M.; Nicoletti, F.; Brunello, N.; Drago, F.; Sortino, M. A.; Copani, A. Fluoxetine Prevents Aβ1-42-Induced Toxicity via a Paracrine Signaling Mediated by Transforming-Growth-Factor-B1. *Front Pharmacol* **2016**, *7*, 389. https://doi.org/10.3389/fphar.2016.00389.

(4) Copani, A.; Condorelli, F.; Caruso, A.; Vancheri, C.; Sala, A.; Giuffrida Stella, A. M.; Canonico, P. L.; Nicoletti, F.; Sortino, M. A. Mitotic Signaling by Beta-Amyloid Causes Neuronal Death. *FASEB J* **1999**, *13* (15), 2225–2234.

(5) Garcia, M. C. The Effect of the Mobile Phase Additives on Sensitivity in the Analysis of Peptides and Proteins by High-Performance Liquid Chromatography–Electrospray Mass Spectrometry. *Journal of Chromatography B* **2005**, *825* (2), 111–123.

(6) Grasso, G.; D’Agata, R.; Rizzarelli, E.; Spoto, G.; D’Andrea, L.; Pedone, C.; Picardi, A.; Romanelli, A.; Fragai, M.; Yeo, K. J. Activity of Anchored Human Matrix Metalloproteinase-1 Catalytic Domain on Au (111) Surfaces Monitored by ESI-MS. *Journal of mass spectrometry* **2005**, *40* (12), 1565–1571.

(7) Grasso, G.; Bush, A. I.; D’Agata, R.; Rizzarelli, E.; Spoto, G. Enzyme Solid-State Support Assays: A Surface Plasmon Resonance and Mass Spectrometry Coupled Study of Immobilized Insulin Degrading Enzyme. *European biophysics journal* **2009**, *38* (4), 407–414.

(8) Teramura, Y.; Iwata, H. Label-Free Immunosensing for α-Fetoprotein in Human Plasma Using Surface Plasmon Resonance. *Analytical biochemistry* **2007**, *365* (2), 201–207.

(9) Piliarik, M.; Bocková, M.; Homola, J. Surface Plasmon Resonance Biosensor for Parallelized Detection of Protein Biomarkers in Diluted Blood Plasma. *Biosensors and Bioelectronics* **2010**, *26* (4), 1656–1661.
